# Impaired self-awareness of cognitive deficits in Parkinson's disease relates to cingulate cortex dysfunction

**DOI:** 10.1017/S0033291721002725

**Published:** 2023-03

**Authors:** Franziska Maier, Andrea Greuel, Marius Hoock, Rajbir Kaur, Masoud Tahmasian, Frank Schwartz, Ilona Csoti, Frank Jessen, Alexander Drzezga, Thilo van Eimeren, Lars Timmermann, Carsten Eggers

**Affiliations:** 1 Department of Psychiatry and Psychotherapy, Medical Faculty and University Hospital Cologne, University of Cologne, Cologne, Germany; 2 Department of Neurology, University Hospital Giessen and Marburg, Marburg, Germany; 3 Institute of Medical Science and Technology, Shahid Beheshti University, Tehran, Iran; 4 Department of Neurology, Hospital of the Brothers of Mercy, Trier, Germany; 5 Gertrudis Clinic, Parkinson-Center, Leun-Biskirchen, Germany; 6 German Center for Neurodegenerative Disorders (DZNE), Bonn, Germany; 7 Department of Nuclear Medicine, Medical Faculty and University Hospital Cologne, University of Cologne, Cologne, Germany; 8 Cognitive Neuroscience, Institute of Neuroscience and Medicine (INM-2), Research Center Jülich, Jülich, Germany; 9 Department of Neurology, Medical Faculty and University Hospital Cologne, University of Cologne, Cologne, Germany; 10 Cognitive Neuroscience, Institute of Neuroscience and Medicine (INM-3), Research Center Jülich, Jülich, Germany; 11 Center for Mind, Brain and Behavior (CMBB), Universities of Marburg and Giessen, Giessen and Marburg, Germany

**Keywords:** Anosognosia, impaired self-awareness, mild cognitive impairment, multimodal neuroimaging, Parkinson's disease

## Abstract

**Background:**

Impaired self-awareness of cognitive deficits (ISAcog) has rarely been investigated in Parkinson's disease (PD). ISAcog is associated with poorer long-term outcome in other diseases. This study examines ISAcog in PD with and without mild cognitive impairment (PD-MCI), compared to healthy controls, and its clinical-behavioral and neuroimaging correlates.

**Methods:**

We examined 63 PD patients and 30 age- and education-matched healthy controls. Cognitive state was examined following the Movement Disorder Society Level II criteria. ISAcog was determined by subtracting *z*-scores (based on controls' scores) of objective tests and subjective questionnaires. Neural correlates were assessed by structural magnetic resonance imaging (MRI) and 2-[fluorine-18]fluoro-2-deoxy-d-glucose-positron emission tomography (FDG-PET) in 47 patients (43 with MRI) and 11 controls. We analyzed whole-brain glucose metabolism and cortical thickness in regions where FDG-uptake correlated with ISAcog.

**Results:**

PD-MCI patients (*N* = 23) showed significantly more ISAcog than controls and patients without MCI (*N* = 40). When all patients who underwent FDG-PET were examined, metabolism in the bilateral superior medial frontal gyrus, anterior and midcingulate cortex negatively correlated with ISAcog (FWE-corrected p < 0.001). In PD-MCI, ISAcog was related to decreased metabolism in the right superior temporal lobe and insula (*N* = 13; FWE-corrected *p* = 0.023) as well as the midcingulate cortex (FWE-corrected *p* = 0.002). Cortical thickness was not associated with ISAcog in these regions. No significant correlations were found between ISAcog and glucose metabolism in controls and patients without MCI.

**Conclusions:**

Similar to Alzheimer's disease, the cingulate cortex seems to be relevant in ISAcog in PD. In PD-MCI patients, ISAcog might result from a disrupted network that regulates awareness of cognition and error processes.

## Introduction

Syndromes of reduced awareness of motor- and non-motor symptoms in patients with Parkinson's disease (PD) have raised increased interest in recent years. While anosognosia (defined as complete unawareness of deficits) has been rarely reported in PD, a partial loss of insight, termed impaired self-awareness (ISA), might exist for motor, cognitive, and behavioral symptoms (Maier & Prigatano, 2017). Both complete and partial lack of insight has been associated with reduced patient adherence and increased mortality in other diseases (Koltai, Welsh-Bohmer, & Schmechel, 2001; Turró-Garriga et al., 2013).

Most reports are available for ISA of PD motor symptoms, especially for levodopa-induced dyskinesia (Amanzio et al., 2010, 2014; Pietracupa et al., 2013), but also for hypokinetic motor deficits (Maier et al., 2015, 2012, 2016). A clear neuropsychological correlate for ISA of motor symptoms has not yet been identified (Maier & Prigatano, 2017), although cognitive impairments such as executive dysfunctions (Amanzio et al., 2014) or worse global cognitive state have been suggested (Jenkinson, Edelstyn, Stephens, & Ellis, 2009). While ISA of dyskinesia was associated with longer disease duration, higher levodopa equivalent daily doses (LEDD) and increased glucose metabolism in the bilateral midcingulate cortex, ISA of hypokinesia was related to older age, predominantly left sided hemibody symptoms and decreased glucose metabolism in the right inferior frontal gyrus and insula (Maier et al., 2016).

ISA of cognitive deficits (ISAcog) has been rarely investigated in PD. Traditionally, patients with PD were thought to be fully aware of their cognitive difficulties, especially when compared to patients with Alzheimer's disease (AD) who often have trouble recognizing their cognitive impairments (Maier & Prigatano, 2017; Starkstein et al., 1996). However, recent reports showed ISAcog in PD for executive dysfunction and memory deficits (Kudlicka, Clare, & Hindle, 2013; Lehrner et al., 2015; Orfei et al., 2018). Since the diagnostic criteria for mild cognitive impairment (MCI) in PD by the Movement Disorder Society (MDS) have enabled a more precise classification of cognitive deficits (Litvan et al., 2012), ISAcog was reported in 16% of PD patients with mild cognitive impairment (PD-MCI) by assessing questionnaires in patients and caregivers (Orfei et al., 2018). Higher ISAcog was associated with lower depression (Orfei et al., 2018).

More research exists for anosognosia or ISAcog in AD, where multiple clinical correlates such as dementia severity, more apathy or less severe depression, and higher caregiver burden were found (Starkstein, 2014). Notably, anosognosia of cognitive deficits in MCI might be an independent predictor for the development of AD (Gerretsen et al., 2017). Numerous neuroimaging correlates have been identified for ISAcog in MCI and Alzheimer's dementia, including changes in regional brain metabolism (Gerretsen et al., 2017; Guerrier et al., 2018), atrophy (Guerrier et al., 2018) and amyloid-deposition (Vannini et al., 2017a). By applying 2-[fluorine-18]fluoro-2-deoxy-d-glucose-positron emission tomography (FDG-PET), imaging studies repeatedly found reduced glucose metabolism in cortical midline structures including the cingulum and prefrontal regions (dorsolateral and orbitofrontal cortex) (Gerretsen et al., 2017; Guerrier et al., 2018; Nobili et al., 2010; Perrotin et al., 2015). Cortical midline regions have been associated with self-appraisal and self-referential processes (Northoff et al., 2006). A neural disconnection between the orbitofrontal cortex (OFC) and the posterior cingulate cortex (PCC) on the one hand and the medial temporal lobe on the other hand has been postulated to contribute to unawareness of memory deficits in AD (Perrotin et al., 2015). Others suggested atrophy and hypometabolism in the dorsal anterior cingulate cortex to be related to cognitive anosognosia (Guerrier et al., 2018).

Cognitive deficits occur early in the course of PD, and can be measurable before the criteria for PD-MCI are fulfilled (Pasquini et al., 2019; Weintraub, Tröster, Marras, & Stebbins, 2018). Widespread reductions in cortical metabolism are also present in early stages (Borghammer et al., 2010), and a specific pattern associated with cognitive dysfunction is partly characterized by hypometabolism in regions belonging to the default mode network, similar to hypometabolism seen in AD (Huang et al., 2007). Awareness of cognitive deficits in PD has not been examined with neuroimaging.

The aim of this study was to further expand our knowledge on ISAcog in PD by (a) comparing awareness of cognitive abilities between healthy controls and patients with PD, (b) analyzing behavioral correlates and the effect of cognitive status (PD-MCI or normal cognition) on ISAcog in PD, and (c) examining the neural correlates of ISAcog. Controls' data were used to determine normal ranges and differentiate between effects of PD and cognitive status. For the first time, we examined neural correlates of ISAcog by applying FDG-PET, followed by cortical thickness analysis to investigate whether associations between metabolism and ISAcog were accompanied by structural changes in the same cortical regions. Our hypotheses were to find ISA in PD patients, negative correlations between glucose metabolism in midline cortical regions and ISAcog, and potentially differences in neural correlates of ISAcog between patients with and without PD-MCI.

## Methods

### Sample

Sixty-three PD patients and 30 age- and education-matched healthy controls were examined from 2014 to 2016. Patients were recruited from an outpatient movement disorders clinic (Department of Neurology, University Hospital of Cologne, Germany) or a specialized PD rehabilitation center (Gertrudis Klinik, Leun-Biskirchen, Germany). Controls were recruited via advertisements and postings at the University of Cologne.

PD was diagnosed according to the Queen's Square Brain Bank criteria (Hughes, Lees, Daniel, & Blankson, 1993). Exclusion criteria for patients and controls were clinical dementia according to the MDS criteria (Emre et al., 2007), depression [Beck Depression Inventory-2 score > 21, BDI-2 (Beck, Steer, & Brown, 1996)], and other neurological or psychiatric diseases. All patients received an MRI scan to exclude signs of atypical parkinsonian syndromes, relevant structural lesions or severe atrophy. Antiparkinsonian medication had to be stable for the last 4 weeks prior to and throughout study participation. Patients who underwent neurosurgery or patients with severe motor impairment during medication OFF-state [Hoehn & Yahr stage 5 (Martinez-Martin, 2010)] were excluded.

We assert that all procedures contributing to this study comply with the ethical standards of the relevant national and institutional committees on human experimentation (ethical approval numbers 15-325 and 12-265) and with the Helsinki Declaration of 1975, as revised in 2008. All study subjects gave written informed consent prior to participation.

### Clinical rating scales

Motor impairment was measured with the Unified PD Rating Scale-III [UPDRS-III (Fahn, Marsden, Calne, & Goldstein, 1987)] in the medication OFF-state, after at least 12 h discontinuation of levodopa, amantadine and MAO-inhibitors, and 72 h withdrawal from dopamine agonists. The LEDD was calculated (Tomlinson et al., 2010).

### Neuropsychological tests

Following the MDS diagnostic criteria for PD-MCI (Litvan et al., 2012), at least two tests in each of the five domains – attention, memory, language, executive functions, and visual-spatial abilities – were assessed. PD-MCI was diagnosed when either two different tests within the same domain or two different tests amongst different domains were ⩾1.5 standard deviations (s.d.) below the mean of age- and education-corrected norms.

The neuropsychological test battery included the Mini Mental State Examination [MMSE (Folstein, Folstein, & McHugh, 1975)], the Parkinson Neuropsychometric Dementia Assessment [PANDA (Kalbe et al., 2008)], the Boston Naming Test [BNT (Kaplan, Goodglass, & Weintraub, 2001)], the digit span test forwards and backwards [Wechsler Memory Scale (von Aster, Neubauer, & Horn, 2006)], the Regensburger verbal fluency task [alternating fluency sports-fruits, semantic fluency animals (Aschenbrenner, Tucha, & Lange, 2000)], and the modified Wisconsin Card Sorting Test [mWCST, errors (Heaton, 1993)].

The tests were assigned to the domains as follows: attention (digit span forwards, digit span backwards), executive functions (mWCST errors, alternating fluency sports-fruits), language (BNT, semantic fluency animals), memory (subtest delayed recall, PANDA; subtest delayed recall, MMSE), and visual-spatial abilities (subtest cubes, PANDA; subtest pentagons, MMSE). Cognitive tests were performed in the medication ON-state within 2 days of the PET scan.

### Evaluation of cognitive state

All test results were converted to standardized *z*-scores, controlled for age and education. In case of the absence of norms, *z*-scores were calculated according to mean and s.d. of the age- and education-matched controls. This concerned the BNT, and the PANDA and MMSE subtests. According to the 10 *z*-scores (two per domain), patients were classified as either PD with normal cognition (PD-NC) or PD-MCI (Litvan et al., 2012). Additionally, an overall mean cognition *z*-score was calculated.

### Assessment of self-awareness

The Cognitive Failures Questionnaire [CFQ (Klumb, 1995)] was used to assess subjective cognitive failures in everyday life. It contains 25 questions, each rated from 0 to 4 (never to very often) with a total range of 0–100. Higher scores reflect more cognitive failures. On the basis of the controls' mean total score and s.d., *z*-scores were calculated for all patients and controls. The individual *z*-score was multiplied by −1 to adjust the direction, so that higher scores reflect better cognition and vice versa. Finally, to determine ISAcog, the CFQ *z*-score was subtracted from the overall cognition-*z*-score. Negative values indicate that subjectively perceived cognition (CFQ *z*-score) was better than objectively tested cognition (overall cognition *z*-score), therefore reflecting ISAcog. The ISAcog value represents a dimensional score without a specific cut-off. Similar strategies to assess ISAcog have been used before (Vannini et al., 2017b).

### Statistical analysis of behavioral data

First, all 63 PD patients and 30 age- and education-matched healthy controls were compared concerning demographic and clinical data using IBM SPSS version 25. Based on these matched samples, *z*-scores of cognitive subtest were calculated and the MCI classification was determined. For the following group comparisons, level of significance was set at < 0.05. Non-parametric data were compared applying the Mann–Whitney *U*-test or the Kruskall–Wallis *H*-test in case of three groups. Post-hoc *U* tests were applied and corrected for the number of comparisons (0.05/3 = *p* < 0.0167). Categorical data were compared using the chi-squared test or the Fisher's exact test when two groups were compared. Non-parametric Spearman correlations were conducted to find significant associations between ISAcog and clinical data. The same comparisons were performed in the FDG-PET subsample.

### FDG-PET and MRI data acquisition

FDG-PET scans were acquired in 47 PD patients and 11 controls after overnight fasting in the OFF-state since it has been shown that the ON-state could alter neural functions in PD (Tahmasian et al., 2015, 2017). Images were recorded on a Siemens high-resolution research tomograph (ECAT HRRT) with 207 transaxial image planes and a voxel size of 1.219 mm (isotropic) in 3D acquisition mode. Subjects lay comfortably in a supine position in a quiet room with dimmed light; a vacuum cushion was used to restrict head motion. Following a transmission scan for attenuation correction, 185 MBq 2-[fluorine-18]fluoro-2-deoxy-d-glucose were injected. Recording started 20 s after injection and continued for 60 min, with one frame saved every 10 min. Using software VINCI (Vollmar et al., 2003), frames were co-registered together; an average of frames 3–6 (minute 20–60) was generated and saved in the Nifti format for the following analysis.

High-resolution T1-weighted MRI scans, acquired on a 3T Siemens Magnetom Prisma with a voxel size of 0.9 × 0.9 × 0.9 mm^3^, were available for 43 of the 47 patients and the 11 controls who underwent FDG-PET. In PD patients, MRI scans were performed under regular dopaminergic medication to reduce head motion (Tahmasian et al., 2015).

### FDG-PET data pre-processing

FDG-PET data were pre-processed and analyzed using SPM12 (www.fil.ion.ucl.ac.uk/spm/software/spm12) in Matlab (MathWorks, Inc.). The averaged FDG-PET images of frame 3–6 were first aligned horizontally along the anterior and posterior commissure and co-registered to structural MRIs. Spatial normalization to MNI space was performed using the FDG-PET template (available as an SPM extension), while keeping the original voxel size. Normalized image dimensions were 128/155/128 (*x*/*y*/*z*). For spatial smoothing, a 6 mm full-width at half-maximum (FWHM) Gaussian filter was applied, with filter size selected according to the high-spatial resolution.

### Analysis of FDG-PET and MRI data

Negative and positive correlation analyses between ISAcog and whole-brain FDG metabolism were conducted in each group separately (controls, all PD patients, PD-NC, and PD-MCI) by applying voxel-wise regression analyses in SPM 12 (Berti et al., 2010). Global normalization by proportional scaling was used with default parameters. In controls, the regression analysis was controlled for age, sex, and BDI-2. In patients, all regression analyses were controlled for age, sex, BDI-2, UPDRS-III, and LEDD. Disease duration was not included because of high collinearity with UPDRS-III, and motor symptom severity was deemed more important as a covariate. The resulting map of *T*-values was thresholded at an uncorrected significance level of *p* < 0.005. Only results with a cluster-level FWE-corrected *p* value < 0.05 are reported.

Mean uptake values in regions that showed a significant association with ISAcog were extracted from individual scans with the SPM toolbox MarsBaR (http://marsbar.sourceforge.net), using significant clusters as volumes of interest (VOIs). Scatter plots and correlation graphs of metabolism and ISAcog were created for each VOI, and partial correlations were run to further distinguish between the neural correlates of ISAcog in each subgroup.

To verify that results were specific for ISAcog, the same analysis was performed with overall cognition *z*-scores.

As hypometabolism in neurodegeneration typically precedes structural atrophy (Yau et al., 2015), regions where metabolism correlated negatively with ISAcog were further examined using T1-weighted MRI scans: cortical thickness was estimated using the SPM12-based computational anatomy toolbox [CAT12 (Dahnke, Yotter, & Gaser, 2013)] with default settings (15 × 15 × 15 mm^3^ FWHM Gaussian smoothing, merging of hemispheres and quality control). A vertex-wise correlation analysis was performed with the search area restricted to regions found in the FDG analysis. The automated anatomical labeling (AAL) atlas (Tzourio-Mazoyer et al., 2002) was used to identify anatomical regions that overlapped with clusters found in the FDG-PET voxel-wise regression analysis, these atlas regions were combined using the WFU pickatlas tool and converted to surface coordinates to create a mask for cortical thickness analysis (Maldjian, Laurienti, & Burdette, 2004). The same covariates were included and the same thresholds applied as in the FDG-PET analysis.

## Results

### Demographic, clinical and neuropsychological data

Demographic and clinical information for all patients and controls is shown in Table 1. Results for the FDG-PET group are depicted in the supplementary material (online Supplementary Table S1). The behavioral data described here refer to the whole sample, but were in a comparable range in the PET subsample. Age, education years and MMSE were similar between patients and controls. There were significantly more women in the control group (*N* = 18) than among patients (*N* = 20; *p* = 0.013). Patients had significantly higher BDI-2 values and significantly lower PANDA-scores. The overall cognition *z*-score was significantly lower in patients compared to controls.

**Table 1.** Comparison of demographic and clinical data between age- and education-matched healthy controls and all PD patients

	Controls *N* = 30		PD patients *N* = 63			*p*
	Mean ± s.d.	Median (range)	Mean ± s.d.	Median (range)		
Age (years)	68.83 ± 8.30	70 (51–81)	68.03 ± 9.13	68 (41–86)	*U* = 902.50	0.726
Gender (m/f)	12/18		43/20		χ^2^ = 6.219	**0.013**
Education (years)	14.57 ± 3.03	14.50 (10–18)	14.30 ± 2.76	14 (10–18)	*U* = 890.50	0.649
Disease duration (years)	NA		5.94 ± 4.45	5 (0.5–19)		
UPDRS-III	NA		28.62 ± 11.09	26 (7–54)		
LEDD	NA		470.47 ± 222.15	447.5 (40–945)		
BDI-2	3.83 ± 3.72	3 (0–14)	8.31 ± 5.30	7 (0–20)	*U* = 447.00	**<0.001**
MMSE	28.50 ± 1.43	29 (24–30)	27.92 ± 2.20	29 (21–30)	*U* = 845.00	0.397
PANDA	26.10 ± 2.95	26.50 (19–30)	21.16 ± 6.28	22 (8–30)	*U* = 498.00	**<0.001**
CFQ	23.80 ± 12.68	23 (0–49)	26.83 ± 11.18	28 (0–51)	*U* = 799.50	0.232
Overall cognition *z*-score	0.32 ± 0.43	0.37 (−0.78 to 1.06)	−0.37 ± 0.69	−0.26 (−2.38 to 0.81)	*U* = 344.00	**<0.001**
CFQ *z*-score	0.00 ± 1.53	0.10 (−3.04 to 2.87)	−0.36 ± 1.35	−0.51 (−3.28 to 2.87)	*U* = 799.50	0.232
ISAcog	0.32 ± 1.47	0.33 (−1.96 to 3.63)	−0.01 ± 1.52	0.09 (−3.45 to 3.60)	*U* = 849.00	0.430

s.d., standard deviation; PD, Parkinson's disease; UPDRS-III, Unified Parkinson's disease Rating Scale; LEDD, levodopa equivalent daily dose; BDI-2, Beck Depression Inventory-2; MMSE, Mini Mental State Examination; PANDA, Parkinson Neuropsychometric Dementia Assessment; CFQ, Cognitive Failures Questionnaire; ISAcog, impaired self-awareness of cognitive deficits.

Of the 63 PD patients, 23 (36.5%) had MCI and 40 (63.5%) had normal cognition (see Table 2). Significant group differences between controls, PD-NC, and PD-MCI were found for age, sex, BDI-2, MMSE, and PANDA scores. Post-hoc *U* test showed that PD-MCI patients were significantly older than PD-NC, more depressed than controls and cognitively more impaired (MMSE, PANDA) compared to controls and PD-NC patients. Controls were significantly less depressed than PD-NC. Disease-related data demonstrated that PD-MCI were significantly more impaired on the UPDRS-III and had a significantly higher LEDD than PD-NC. Disease duration was not significantly different between both patient subgroups.

**Table 2.** Group comparison of demographic, clinical and disease related data between healthy controls, PD-NC, and PD-MCI

	Controls *N* = 30		PD-NC *N* = 40		PD-MCI *N* = 23			*p*
	Mean ± s.d.	Median (range)	Mean ± s.d.	Median (range)	Mean ± s.d.	Median (range)		
Age (years)	68.83 ± 8.30	70 (51–81)	65.55 ± 8.82^£^	66.5 (41–83)	72.35 ± 8.13^£^	75 (54–86)	*H* = 9.627	**0.008**
Gender (m/f)	12/18^¥^		30/10^¥^		13/10		χ^2^ = 8.777	**0.012**
Education (years)	14.57 ± 3.03	14.50 (10–18)	14.78 ± 2.81	15 (10–18)	13.48 ± 2.54	13 (11–18)	*H* = 2.960	0.228
Disease duration (years)	NA		5.28 ± 3.88	4 (1–15)	7.11 ± 5.20	6 (0.5–19)	*U* = 367.50	0.185
UPDRS-III	NA		24.79 ± 9.10	23 (7–45)	35.41 ± 11.24	34 (10–54)	*U* = 191.50	**<0.001**
LEDD	NA		402.36 ± 218.26	415 (40–945)	568.20 ± 192.72	500 (250–925)	*U* = 231.00	**0.013**
BDI-2	3.83 ± 3.72^¥,§^	3 (0–14)	8.48 ± 4.90^¥^	7 (0–17)	8.00 ± 6.06^§^	7.5 (0–20)	*H* = 15.240	**<0.001**
MMSE	28.50 ± 1.43^§^	29 (24–30)	28.93 ± 1.29^£^	29 (23–30)	26.17 ± 2.39^§,£^	26 (21–29)	*H* = 24.883	**<0.001**
PANDA	26.10 ± 2.95^§^	26.50 (19–30)	24.80 ± 3.75^£^	25 (17–30)	14.83 ± 4.49^§,£^	14 (8–25)	*H* = 44.921	**<0.001**
CFQ	23.80 ± 12.68	23 (0–49)	27.15 ± 10.55	27.5 (6–47)	26.26 ± 12.42	28 (0–51)	*H* = 1.470	0.480
Overall cognition *z*-score	0.32 ± 0.43^¥,§^	0.37 (−0.78 to 1.06)	0.04 ± 0.36^¥,£^	0.10 (−0.71 to 0.81)	−1.08 ± 0.52^§,£^	−0.94 (−2.38 to −0.36)	*H* = 53.309	**<0.001**
CFQ *z*-score	0.00 ± 1.53	0.10 (−3.04 to 2.87)	−0.40 ± 1.27	−0.45 (−2.80 to 2.15)	−0.30 ± 1.50	−0.51 (−3.28 to 2.87)	*H* = 1.470	0.480
ISAcog	0.32 ± 1.47^§^	0.33 (−1.96 to 3.63)	0.44 ± 1.35^£^	0.54 (−2.29 to 3.60)	−0.79 ± 1.51^§,£^	−0.48 (−3.45 to 2.05)	*H* = 9.770	**0.008**

s.d., standard deviation; PD, Parkinson's disease; NC, normal cognition; MCI, mild cognitive impairment; BDI-2, Beck Depression Inventory-2; MMSE, Mini Mental State Examination; PANDA, Parkinson Neuropsychometric Dementia Assessment; UPDRS-III, Unified Parkinson's Disease Rating Scale-III; LEDD, levodopa equivalent daily dose; CFQ, Cognitive Failures Questionnaire; ISAcog, impaired self-awareness of cognitive deficits.

Post-hoc *U* test: ^¥^ significant difference between controls and PD-NC; ^§^ significant difference between controls and PD-MCI; ^£^ significant difference between PD-NC and PD-MCI.

All 10 neuropsychological tests showed significant differences between the three groups (see online Supplementary Table S3). Of the PD-MCI patients, three (13%) had single-domain MCI (amnestic subtype) and 20 (87%) had multiple-domain MCI. PD-MCI patients were significantly more impaired than controls in all five domains. PD-NC showed significantly more impairment in attention, executive functions, and language compared to controls. Finally, the PD-MCI group was significantly more impaired than PD-NC in visual-spatial abilities, memory, executive functions, and attention. The overall cognition *z*-score was significantly different between all three groups.

### ISAcog

The CFQ was not different between any of the groups and subgroups (controls, all PD patients, PD-NC and PD-MCI). However, when the CFQ self-rating *z*-score was subtracted from the mean overall cognition *z*-score, PD-MCI patients had significantly more ISAcog than PD-NC and controls. ISAcog was similar between controls and PD-NC. Correlation analyses between ISAcog and clinical data can be found in online Supplementary Table S4. Higher ISAcog was significantly associated with less depression (BDI-2, *r* = 0.300, *p* = 0.018) and more impaired executive functions, memory, language and visual-spatial abilities within all PD patients. However, there were no significant correlations between ISAcog and cognitive domains or clinical data in PD-MCI.

### FDG-PET findings

**Fig. 1.**
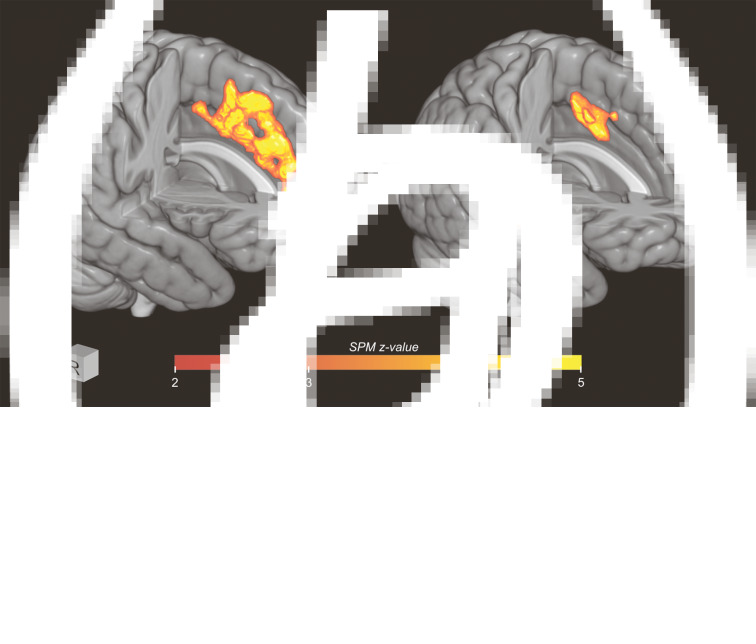
Association of ISAcog and reduced FDG uptake in PD patients. In all Parkinson’ disease patients (a), ISAcog was associated with lower metabolic activity in a cluster encompassing the bilateral superior medial frontal (mPFC), anterior (ACC) and midcingulate cortex (MCC) and supplementary motor area (SMA; *p* < 0.001). When including only patients with MCI (b), in addition to the bilateral MCC and SMA (*p* = 0.002), ISAcog was associated with decreased activity in the right superior temporal and insular cortex (*p* = 0.023). 3D view from right (R), color bar indicates *z* values.

Of 47 patients with FDG-PET, 34 (72.3%) had normal cognition and 13 (27.7%) had PD-MCI (see online Supplementary Table S2). Findings for the voxel-wise correlation analyses are presented in Fig. 1 and Table 3. There was no significant positive or negative correlation between ISAcog and FDG metabolism in controls. In all PD patients, there was a significant correlation between ISAcog and decreased FDG metabolism in a large midline cluster encompassing the bilateral superior medial frontal gyrus (mPFC), anterior (ACC) and midcingulate cortex (MCC) and supplementary motor area (SMA) (FWE-corrected *p* < 0.001). In PD-NC, a cluster in the ACC and mPFC did not pass the FWE-corrected threshold for significance. Finally, two significant clusters were identified in PD-MCI: there was a correlation between ISAcog and decreased metabolism in the bilateral MCC and SMA, overlapping with the posterior part of the cluster seen in the whole PD group (FWE-corrected *p* = 0.002), as well as the right superior temporal lobe and the adjacent right insula (FWE-corrected *p* = 0.023).

**Table 3.** Voxel-wise correlation between ISAcog and regional FDG metabolism in Parkinson's disease patients

	Anatomical region	L/R	Cluster size (voxel)	statistics		Peak (MNI)		
				*z*	*p*	*x*	*y*	*z*
All PD patients (*N* = 47)	Superior medial frontal cortex	L	5048	4.24	**<0.001**	1	36	35
	Anterior cingulate cortex	R	5048	4.18	**<0.001**	6	42	23
	Supplementary motor area	L	5048	3.98	**<0.001**	−2	8	45
PD-MCI (*N* = 13)	Superior temporal cortex	R	685	4.34	**0.023**	48	−25	4
	Insula	R	685	3.88	**0.023**	35	−27	15
	Superior temporal cortex	R	685	3.00	**0.023**	48	−19	−4
	Midcingulate cortex	R	1004	4.23	**0.002**	5	−3	43
	Midcingulate cortex	R	1004	4.19	**0.002**	5	4	40
	Supplementary motor area	L	1004	3.92	**0.002**	−6	10	43

PD, Parkinson's disease; MCI, mild cognitive impairment; FWE, family-wise error.

Anatomical regions are for peak coordinates in AAL atlas and encompass most voxels per cluster.

*p* values are cluster-level FWE-corrected.

**Fig. 2.**
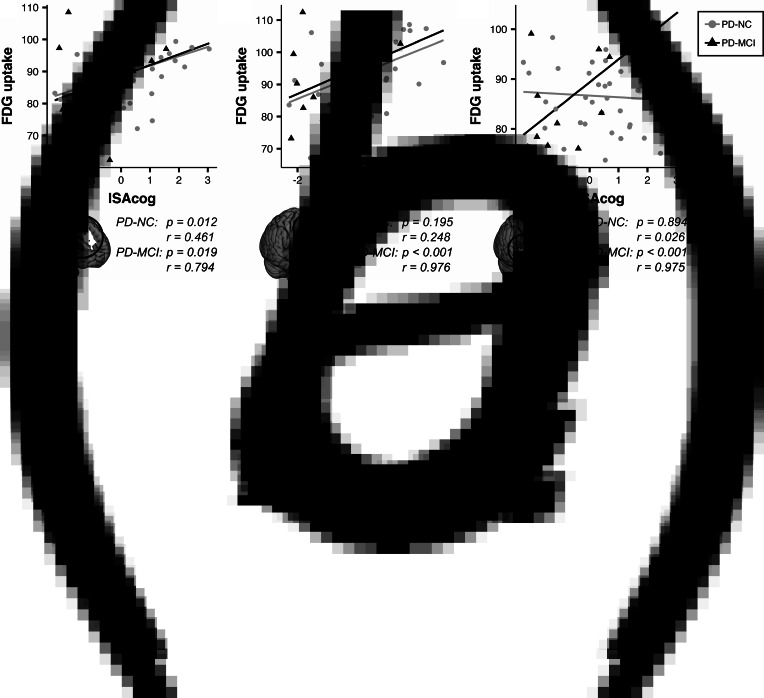
ISAcog and mean cluster FDG uptake values in PD patients. Mean normalized FDG uptake values in clusters found in the voxel-wise analysis with (a) all patients and (b), (c) PD-MCI are plotted against ISAcog. For each region, a partial correlation with the covariates age, gender, LEDD, motor impairment (UPDRS-III) and depression (BDI-2) was performed for patients with normal cognition (PD-NC; gray, circles) and patients with MCI (PD-MCI; black, triangles). Lines represent linear correlations without covariates; statistics (*p* values and partial correlation coefficients) are covariate-adjusted.

The three significant clusters were used as VOIs and subjects' normalized mean uptake in each of these regions was extracted from PET scans. To further investigate potentially distinct mechanisms of ISAcog between PD-NC and PD-MCI, VOI values were correlated with ISAcog separately for PD-NC and PD-MCI, controlling for age, sex, BDI-2, UPDRS-III, and LEDD. ISAcog *z*-scores and mean FDG uptake values in each VOI are plotted in Fig. 2. The association between ISAcog and metabolism in the large cluster detected within the whole PD group was significant in both the PD-NC and PD-MCI subgroups, whereas the correlation in the clusters detected with PD-MCI patients, especially in the right superior temporal lobe and insula, remained specific for this subgroup.

The voxel-wise correlation with overall cognition *z*-scores did not show any significant clusters with all patients and both subgroups. Likewise, when mean uptake values from clusters associated with ISAcog were correlated with objective cognitive scores, no correlations were found in all patients and PD-MCI. Only in PD-NC, mean uptake in the clusters found with all patients also correlated with objective cognition, but this correlation was much weaker than with ISAcog. Thus, the previously described associations are specific for ISAcog and could not be explained by associations between metabolism and cognitive performance.

### Cortical thickness findings

To examine whether ISA was also associated with gray matter structure in the regions found with FDG-PET, cortical thickness was analyzed in 43 PD patients (34 PD-NC and 9 PD-MCI). Again, this step was not performed in controls since FDG-PET analysis did not show any significant association. A vertex-wise regression was performed in AAL regions that overlapped with clusters where metabolism correlated with ISAcog, including the bilateral superior medial frontal gyrus, ACC, MCC and SMA, as well as the right insula, superior temporal gyrus, Heschl gyrus and Rolandic operculum. No correlations were observed that remained significant after correction for multiple comparisons when considering all patients, as well as PD-NC and PD-MCI separately.

## Discussion

This is the first study that examined glucose metabolism and cortical thickness in relation to ISAcog in PD patients and matched controls. We found a significant negative association between reduced metabolism and ISAcog in a large cluster comprising the bilateral superior medial frontal gyrus, ACC, MCC and SMA. When only patients with PD-MCI were considered, this cluster was restricted to the MCC and SMA, while a new region was found in the right superior temporal lobe and adjacent insula. No significant clusters could be identified in healthy controls and PD-NC. A correlation in PD-NC was however measurable by a subsequent VOI analysis for the cluster detected with all patients. Additional analyses of cortical thickness did not show an association between the degree of atrophy and ISAcog in the regions found with FDG-PET. We show that the results are specific for ISAcog, since its correlation with metabolism could not be explained by correlations between FDG-uptake and objective cognition.

First, considering the behavioral outcomes of this study, no differences in ISAcog were found between all patients and education- and age-matched controls. When PD patients were classified into PD-NC and PD-MCI, increased ISAcog was only detected in PD-MCI compared to controls and PD-NC, although both PD-NC and PD-MCI were significantly more impaired in overall cognition than controls. This demonstrates that ISAcog and objective cognition are independent measures and supports the validity of our approach. A similar result has been reported in another study that applied a different method to assess ISAcog in PD (Orfei et al., 2018). Additionally, as indicators of a more progressed disease, PD-MCI patients were significantly older, had higher UPDRS-III-scores, and had a higher LEDD. Significantly worse cognitive test scores in PD-MCI compared to PD-NC were found for the digit span backwards test, alternating verbal fluency, both tests for delayed recall, and visual spatial abilities (pentagons and cubes). However, correlation analyses did not discover significant associations in PD-MCI between ISAcog and the cognitive domains. This finding has been reported by others (Maier et al., 2016; Orfei et al., 2018; Pillai, Bonner-Jackson, Floden, Fernandez, & Leverenz, 2018).

Second, as hypothesized and similar to findings in AD patients (Guerrier et al., 2018; Nobili et al., 2010; Perrotin et al., 2015), reduced glucose metabolism in midline structures, particularly in the cingulate cortex, might play a crucial role in the development of ISAcog in PD. Our study suggests that the exact regions involved depend on patients' cognitive state, with the ACC detected only in the whole group and the role of the MCC being more consistent. When all PD patients were considered, a large cluster comprising the ACC and adjacent frontal superior medial cortex, MCC and SMA was associated with ISAcog. However, when PD-NC and PD-MCI were separated, only in PD-MCI metabolism and ISAcog correlated in the MCC and adjacent SMA. The MCC has been identified as a separate structure, which is not part of the ACC, even though it has often been named ‘dorsal ACC’ (Guerrier et al., 2018). The term ‘dorsal ACC’ instead has been criticized since it implies that the MCC is a subdivision of the ACC (Vogt, 2016). The MCC is activated in tasks that involve information processing and cognitive control, such as error processing (Fiehler, Ullsperger, & Von Cramon, 2004; Holroyd et al., 2004), action/conflict monitoring (Mayr, 2004), and working memory tasks (Petit, Courtney, Ungerleider, & Haxby, 1998). Studies that analyzed anosognosia in early stage AD and AD dementia have found similar results (Guerrier et al., 2018). Recently, Guerrier and colleagues used FDG-PET and structural MRI data and compared patient's self-ratings to proxy-ratings to asses ISAcog (Guerrier et al., 2018). They found significant hypometabolism in the dorsal ACC (equals MCC) in patients with ISAcog, but their neuropsychological test results did not correlate with ISAcog. The authors speculated that a failure in the updating process of one's own database leads to a lack of integration of self-information which might be caused by executive dysfunction and therefore might result in ISAcog. By comparing amnestic MCI patients with higher and lower ISAcog, Nobili et al. (2010) found reduced glucose metabolism in the superior temporal gyrus, the inferior parietal lobule and the angular gyrus in unaware MCI patients applying FDG-PET (Nobili et al., 2010). When a correlation analysis was performed that included the whole patient group, higher ISAcog was related to hypometabolism in the MCC, the PCC, the inferior parietal lobule, the angular gyrus, and the precuneus. The authors speculated that the posteromedial cortex seems to be a key structure in a memory awareness network in AD (Nobili et al., 2010).

A multimodal imaging functional MRI and FDG-PET study by Perrotin et al. (2015) found reduced glucose metabolism in the OFC and PCC in patients with probable AD who showed ISAcog of memory deficits (Perrotin et al., 2015). When OFC and PCC were used as seeds to analyze intrinsic connectivity, higher ISAcog was associated with reduced intrinsic connectivity between OFC and PCC, and also with the medial temporal lobe. The involvement of the medial temporal lobe led to the hypothesis that ISAcog might be a consequence of disturbed communication between and within networks which are involved in self-referential processes and memory functions (Perrotin et al., 2015). We speculate that in our study the association between ISAcog and metabolism in the right superior temporal lobe and adjacent insula might be related to the parallel finding in the MCC, possibly resulting from a disrupted network that regulates awareness of cognitive deficits and error processes. The insula has been shown to be connected with the superior temporal gyrus and the MCC (Ghaziri et al., 2017), and is clearly involved in neural networks that are associated with reduced awareness (Cosentino et al., 2015). These findings were specific for ISAcog in PD-MCI, but it remains unclear if certain cognitive domains are crucial for this distinction.

However, when discussing findings from MCI and AD dementia research, one has to keep in mind that this study examined PD patients. Moreover, most of the included patients had multiple-domain PD-MCI and not single-domain amnestic-MCI. Therefore, the transferability of AD research results is limited. Nevertheless, reduced glucose metabolism in cingulate and temporal regions has been reported in PD-MCI patients compared to PD-NC (Tang et al., 2016). Since we did not examine cerebral spinal fluid amyloid and tau biomarkers, it remains unclear whether some of the included PD patients had a co-existent Alzheimer's pathology which might have contributed to ISAcog. More research is needed to answer this question. Similarly, it would be of interest to include patients with a genetic predisposition for PD-MCI or specific cognitive phenotypes.

Another critical remark relates to the neuropsychological test battery. Due to the large amount of tests and examinations in this study, we decided to choose rather ‘short’ cognitive tests such as subtests of the MMSE to determine Level II PD-MCI instead of applying an extensive battery. This decision was made in order to not exhaust our cognitively impaired patients and ensure they could complete the study protocol without difficulties. Also, the possible influence of apathy on ISAcog was not examined. Finally, the small number of controls who underwent FDG-PET imaging limits our results.

In conclusion, this is the first report of neural correlates for ISAcog in PD. Similarly to AD patients, the cingulate cortex seems to play a crucial role. In PD-MCI, a disrupted network that regulates awareness of cognitive deficits and error processes, encompassing the right superior temporal lobe and adjacent insula as well as the MCC, might be responsible for ISAcog. Future studies applying connectivity analyses are needed to verify this hypothesis.

## Supplementary material

For supplementary material accompanying this paper visit https://doi.org/10.1017/S0033291721002725. click here to view supplementary material
