# Supplementary material for: Impaired self-awareness of cognitive deficits in Parkinson's disease relates to cingulate cortex dysfunction
Source: Psychol Med. 2021 Sep 23;53(4):1244–53. doi: 10.1017/S0033291721002725 (PMC10009405; doi:10.1017/S0033291721002725)
Supplement: Supplementary file 1 [file S0033291721002725sup.zip › S0033291721002713sup001.docx]

**SUPPLEMENTARY MATERIALS 1 - Complete item pool**

**HOME BASED:**

1. **Open all the curtains in your home.**
2. **Look out of a front window for 2mins.**
3. **Sit in a communal/shared area by yourself for 5mins.**
4. **Sit in a communal or shared area with others for 5mins.**
5. **Open the front door and stand in the doorway for 5mins.**
6. **Stand outside your home with someone you know for 5mins.**
7. **Stand outside your home on your own for 5mins.**

**OUTDOORS:**

1. **Walk down a quiet street with someone you know.**
2. **Walk down a quiet street on your own.**
3. **Walk down a busy street with someone you know.**
4. **Walk down a busy street on your own.**
5. **Walk through a park with someone you know for 10mins.**
6. **Walk through a park on your own for 10mins.**
7. **Sit on a bench in a public place, with someone you know for 5mins.**
8. **Sit on a bench in a public place on your own for 5mins.**

**TRANSPORTATION:**

1. **Wait at a bus stop with someone you know for 5mins.**
2. **Travel with someone you know on the bus for one stop.**
3. **Travel with someone you know on the bus for several stops.**
4. **Wait at a bus stop on your own for 5mins.**
5. **Travel on your own on the bus for one stop.**
6. **Travel on your own on the bus for several stops.**
7. **Sit next to a stranger on the bus.**

**APPOINTMENTS:**

1. **Walk into the waiting room of your GP/health centre with someone you know.**
2. **Walk into the waiting room of your GP/health centre on your own.**
3. **Sit in the waiting room of your GP/health centre with someone you know for 5mins.**
4. **Sit in the waiting room of your GP/health centre on your own for 5mins.**
5. **Queue to speak with the receptionist in your GP/health centre.**
6. **See your GP for a health appointment.**

**SHOPPING:**

1. **Go into a local shop with someone you know for 5mins.**
2. **Go into a local shop on your own for 5mins.**
3. **Purchase an item in a local shop, from a shop assistant.**
4. **Go to a shopping centre with someone you know for 15mins.**
5. **Go to a shopping centre on your own for 15mins.**
6. **Speak to a shop assistant in local shopping centre (e.g., ask if they have a specific item in stock?)**

**CAFÉ:**

1. **Go into a café with someone you know.**
2. **Go into a café on your own.**
3. **Sit in a café with someone you know for 10mins.**
4. **Sit in a café on your own for 10mins.**
5. **Order a drink on your own in a cafe.**
6. **Sit near to other people (strangers) in the cafe for 10mins.**

**
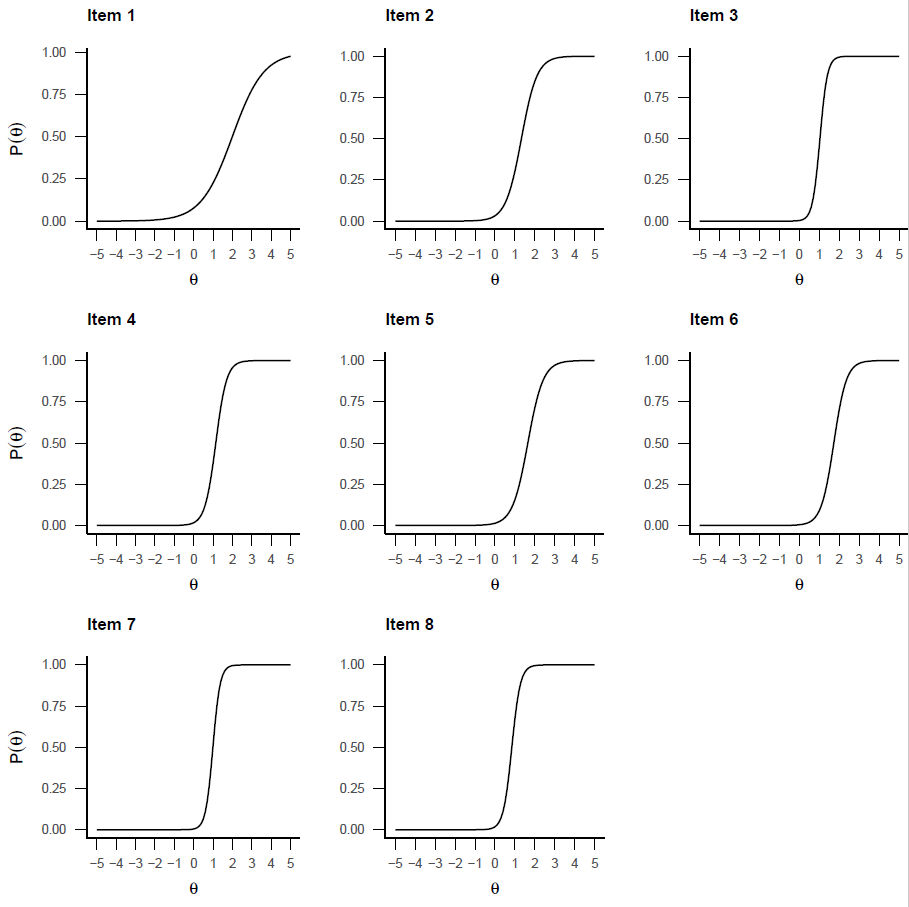
SUPPLEMENTARY MATERIALS 2. Item probability trace lines**

**Figure S1. Item characteristic curves for the binary O-AS avoidance items. Plot lines represent the probability (y axis) of endorsing that item across the theta (i.e. severity) spectrum (x axis).**

**
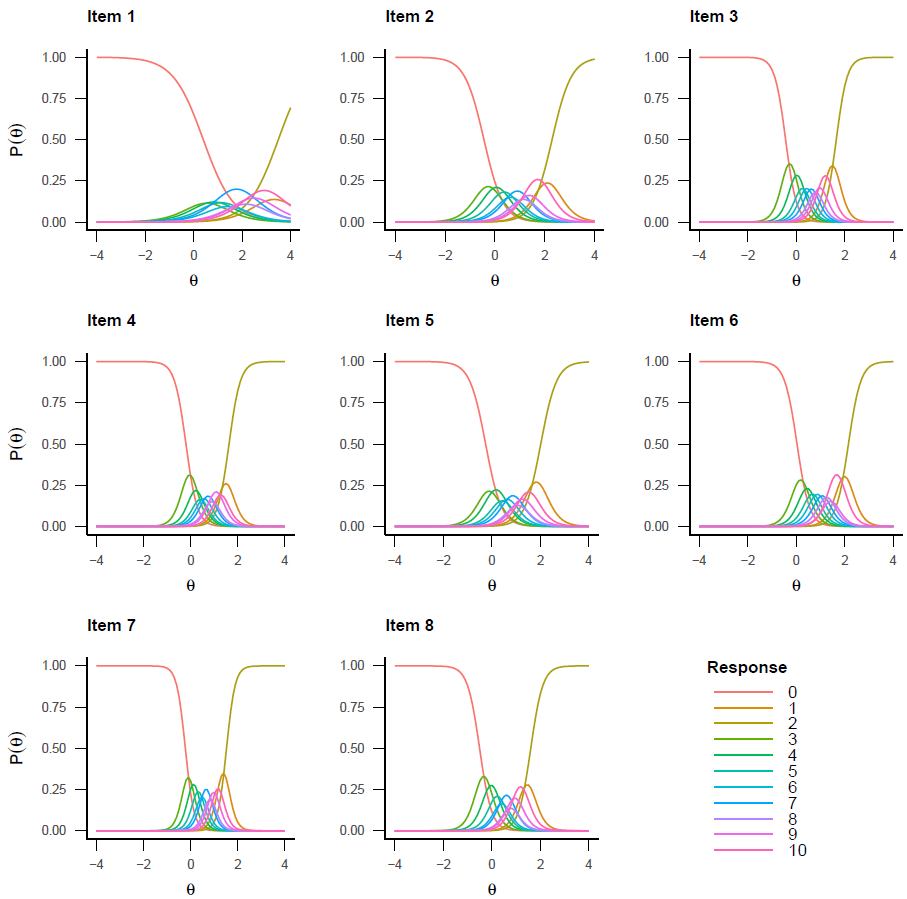
**

**Figure S2. Category response curves for the polytomous O-AS distress items. Plot lines represent the probability (y axis) of responding to each scale option (0-10) across the theta (i.e. severity) spectrum (x axis).**

**SUPPLEMENTARY MATERIALS 3. Item information functions**

**
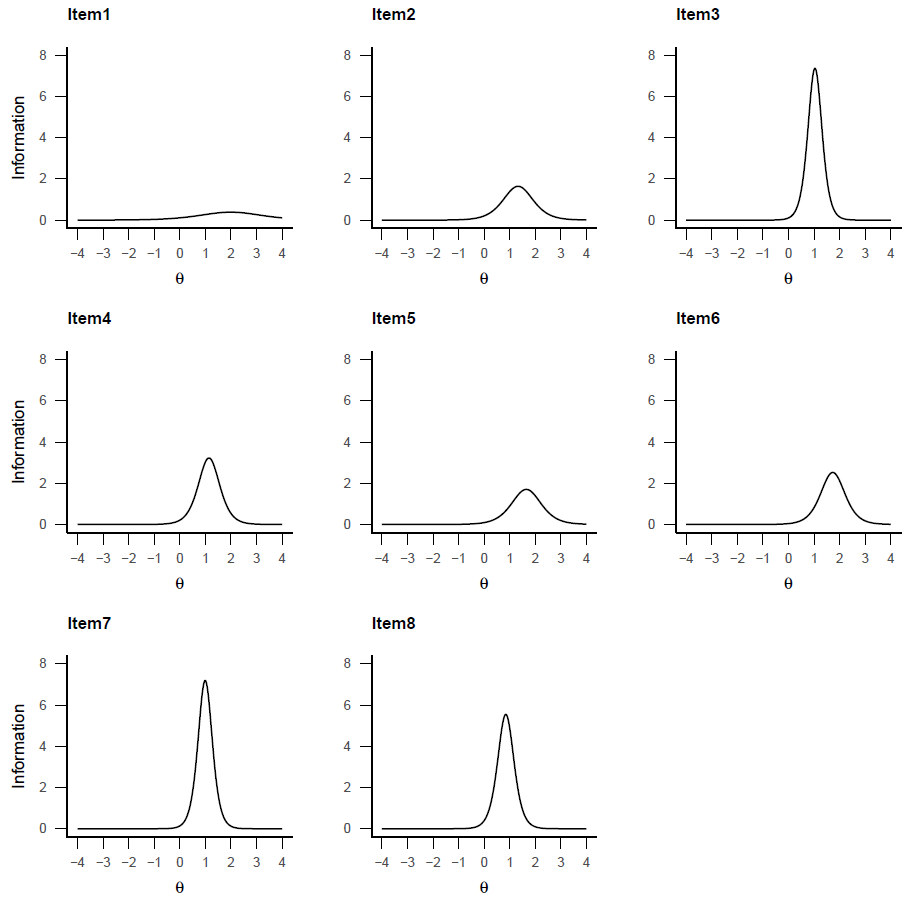
**

**Figure S3. Item information functions for all O-AS avoidance items**

**
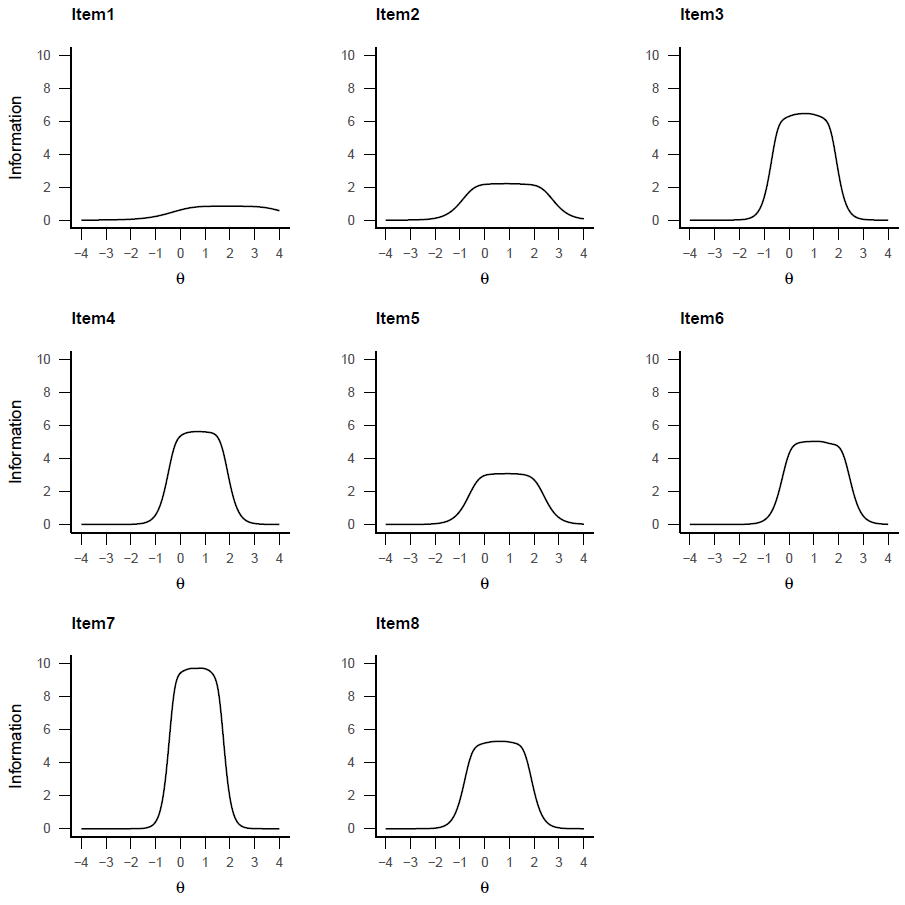
**

**Figure S4. Item information functions for all O-AS distress items**

**SUPPLEMENTARY MATERIALS 4. Receiver Operating Characteristic (ROC) plots**

**
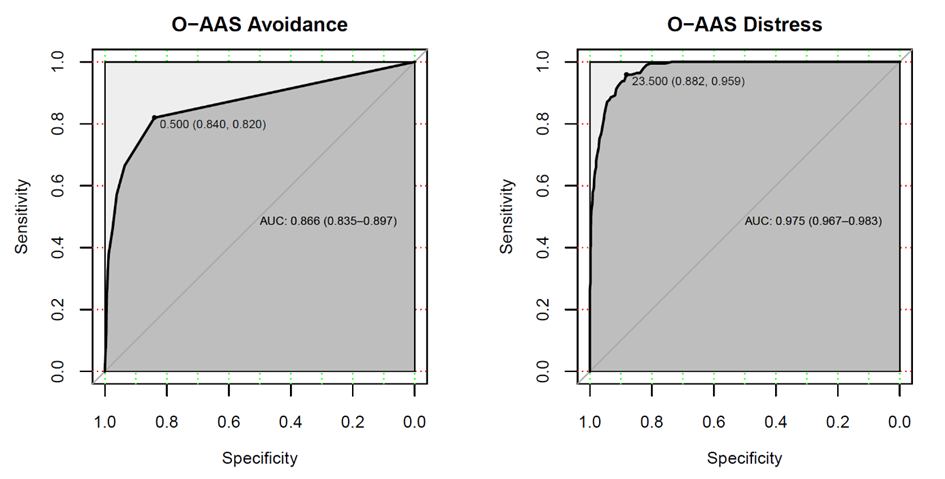
**

**Figure S5. Receiver Operator Curve plots for the O-AS avoidance and distress scores. The area under the curve (AUC) and the optimal threshold for discriminating patients with psychosis and agoraphobia (*n*=194) and controls from the general population (*n*=1094) are displayed.**
