# Supplementary material for: Impaired self-awareness of cognitive deficits in Parkinson's disease relates to cingulate cortex dysfunction
Source: Psychol Med. 2021 Sep 23;53(4):1244–53. doi: 10.1017/S0033291721002725 (PMC10009405; doi:10.1017/S0033291721002725)
Supplement: Supplementary file 1 [file S0033291721002725sup.zip › S0033291721002725sup001.docx]

**Table S1.** Comparison of age- and education-matched healthy controls and PD patients (FDG-PET group).

|  | Controls  N=11 |  | PD patients  N=47 |  | test statistic | p |
| --- | --- | --- | --- | --- | --- | --- |
|  | Mean ± SD | Median (range) | Mean ± SD | Median (range) |  |  |
| Age (yrs) | 64.91 ± 8.48 | 65 (52 – 77) | 67.62 ± 8.29 | 68 (51 – 83) | *U* = 212.00 | 0.356 |
| Gender (m/f) | 4/7 |  | 32/15 |  | *Χ*^2^ = 3.455 | 0.083 |
| Education (yrs) | 16.09 ± 2.39 | 17 (12 – 18) | 14.34 ± 2.84 | 15 (10 – 18) | *U* = 163.00 | 0.054 |
| Disease duration (yrs) | NA |  | 5.07 ± 3.92 | 4 (0.5 – 18) |  |  |
| UPDRS-III | NA |  | 26.33 ± 10.14 | 24 (7 – 46) |  |  |
| LEDD | NA |  | 434.75 ± 215.95 | 420 (40 – 945) |  |  |
| BDI-2 | 2.36 ± 1.96 | 2 (0 – 6) | 8.15 ± 5.28 | 7 (0 – 20) | *U* = 83.00 | **<0.001** |
| MMSE | 29.18 ± 0.87 | 29 (27 – 30) | 28.30 ± 1.91 | 29 (22 – 30) | *U* = 187.50 | 0.143 |
| PANDA | 27.00 ± 2.00 | 27 (23 – 30) | 22.23 ± 5.41 | 23 (8 – 30) | *U* = 111.50 | **0.003** |
| CFQ | 27.82 ± 12.55 | 31 (10 – 49) | 26.43 ± 10.47 | 27 (6 – 46) | *U* = 238.00 | 0.684 |
| Overall Cognition z-score | 0.44 ± 0.29 | 0.44 (-0.02 – 0.96) | -0.26 ± 0.52 | -0.06 (-1.63 – 0.40) | *U* = 51.00 | **<0.001** |
| CFQ z-score | -0.48 ± 1.51 | -0.87 (-3.04 – 1.66) | -0.32 ± 1.26 | -0.39 (-2.68 – 2.15) | *U* = 238.00 | 0.684 |
| ISAcog | 0.92 ± 1.56 | 0.87 (-1.27 – 3.63) | 0.06 ± 1.38 | 0.32 (-2.29 – 3.05) | *U* = 169.00 | 0.076 |

Abbreviations: SD, standard deviation; PD, Parkinson’s disease; UPDRS-III, Unified Parkinson’s disease Rating Scale; LEDD, Levodopa equivalent daily dose; BDI-2, Beck Depression Inventory-2; MMSE, Mini Mental State Examination; PANDA, Parkinson Neuropsychometric Dementia Assessment; CFQ, Cognitive Failures Questionnaire; ISAcog, impaired self-awareness of cognitive deficits.
