# Supplementary material for: Impaired self-awareness of cognitive deficits in Parkinson's disease relates to cingulate cortex dysfunction
Source: Psychol Med. 2021 Sep 23;53(4):1244–53. doi: 10.1017/S0033291721002725 (PMC10009405; doi:10.1017/S0033291721002725)
Supplement: Supplementary file 1 [file S0033291721002725sup.zip › S0033291721002725sup002.docx]

**Table S2.** Group comparison of demographic, clinical and disease related data between healthy controls, PD-NC, and PD-MCI (FDG-PET group).

|  | Controls  N=11 |  | PD-NC  N=34 |  | PD-MCI  N=13 |  |  | p |
| --- | --- | --- | --- | --- | --- | --- | --- | --- |
|  | Mean ± SD | Median (range) | Mean ± SD | Median (range) | Mean ± SD | Median (range) |  |  |
| Age (yrs) | 64.91 ± 8.48 | 65 (52 – 77) | 66.50 ± 7.79 | 66.5 (51 – 83) | 70.54 ± 9.15 | 74 (54 – 82) | *H* = 3.437 | 0.179 |
| Gender (m/f) | 4/7 |  | 25/9 |  | 7/6 |  | *Χ*^2^ = 5.357 | 0.069 |
| Education (yrs) | 16.09 ± 2.39 | 17 (12 – 18) | 14.41 ± 2.81 | 15 (10 – 18) | 14.15 ± 3.02 | 13 (11 – 18) | *H* = 3.745 | 0.154 |
| Disease duration (yrs) | NA |  | 4.79 ± 3.72 | 4 (1 – 15) | 5.81 ± 4.48 | 5 (0.5 – 18) | *U* = 189.00 | 0.444 |
| UPDRS-III | NA |  | 25.06 ± 9.87 | 23 (7 – 45) | 29.83 ± 10.48 | 30 (10 – 46) | *U* = 138.00 | 0.123 |
| LEDD | NA |  | 402.36 ± 218.26 | 415 (40 – 945) | 516.97 ± 193.99 | 435 (250 – 900) | *U* = 158.50 | 0.172 |
| BDI-2 | 2.36 ± 1.96^¥^ | 2 (0 – 6) | 8.32 ± 4.95^¥^ | 7 (0 – 17) | 7.67 ± 6.34 | 6.5 (0 – 20) | *H* = 12.337 | **0.002** |
| MMSE | 29.18 ± 0.87^§^ | 29 (27 – 30) | 28.91 ± 1.36^£^ | 29 (23 – 30) | 26.69 ± 2.25^§, £^ | 27 (22 – 29) | *H* = 15.142 | **0.001** |
| PANDA | 27.00 ± 2.00^§^ | 27 (23 – 30) | 24.41 ± 3.70^£^ | 25 (17 – 30) | 16.54 ± 5.11^§, £^ | 17 (8 – 25) | *H* = 24.156 | **<0.001** |
| CFQ | 27.82 ± 12.55 | 31 (10 – 49) | 26.79 ± 10.32 | 27 (6 – 46) | 25.46 ± 11.21 | 27 (9 – 43) | *H* = 0.278 | 0.870 |
| Overall Cognition z-score | 0.44 ± 0.29^¥,§^ | 0.44 (-0.02 – 0.96) | -0.02 ± 0.32^¥,£^ | 0.05 (-0.71 – 0.40) | -0.89 ± 0.39^§,£^ | -0.77 (-1.63 - -0.36) | *H* = 34.678 | **<0.001** |
| CFQ z-score | -0.48 ± 1.51 | -0.87 (-3.04 – 1.66) | -0.36 ± 1.24 | -0.39 (-2.68 – 2.15) | -0.20 ± 1.35 | -0.39 (-2.31 – 1.78) | *H* = 0.278 | 0.870 |
| ISAcog | 0.92 ± 1.56^§^ | 0.87 (-1.27 – 3.63) | 0.35 ± 1.30 | 0.54 (-2.29 – 3.05) | -0.69 ± 1.35^§^ | -1.12 (-2.22 – 1.56) | *H* = 7.724 | **0.021** |

Abbreviations: SD, standard deviation; PD, Parkinson’s disease; NC, normal cognition; MCI, mild cognitive impairment; BDI-2, Beck Depression Inventory-2; MMSE, Mini Mental State Examination; PANDA, Parkinson Neuropsychometric Dementia Assessment; UPDRS-III, Unified Parkinson’s Disease Rating Scale-III; LEDD, Levodopa equivalent daily dose; CFQ, Cognitive Failures Questionnaire; ISAcog, impaired self-awareness of cognitive defictis. Post-hoc U-test: ^¥^ significant difference between controls and PD-NC; ^§^ significant difference between controls and PD-MCI; ^£^ significant difference between PD-NC and PD-MCI.
