# Supplementary material for: Impaired self-awareness of cognitive deficits in Parkinson's disease relates to cingulate cortex dysfunction
Source: Psychol Med. 2021 Sep 23;53(4):1244–53. doi: 10.1017/S0033291721002725 (PMC10009405; doi:10.1017/S0033291721002725)
Supplement: Supplementary file 1 [file S0033291721002725sup.zip › S0033291721002725sup003.docx]

**Table S3.** Comparison of cognitive test results (z-scores), overall cognition, cognitive failures questionnaire, and ISAcog between controls, PD-NC and PD-MCI.

|  | Controls  N=30 | PD-NC  N=40 | PD-MCI  N=23 |  | p |
| --- | --- | --- | --- | --- | --- |
| Domain: Attention |  |  |  |  |  |
| Digit span forwards | 0.81 ± 0.85^¥,§^ | 0.11 ± 0.94^¥^ | -0.48 ± 0.93^§^ | *H* = 20.37 | **<0.001** |
| Digit span backwards | 0.27 ± 0.93^§^ | -0.11 ± 0.88^£^ | -0.82 ± 0.85^§,£^ | *H* = 17.15 | **<0.001** |
| Domain: Executive functions |  |  |  |  |  |
| WCST errors | 0.00 ± 0.77^¥,§^ | -0.52 ± 0.56^¥^ | -0.84 ± 0.71^§^ | *H* = 18.67 | **<0.001** |
| Alternating fluency | 0.87 ± 0.91^§^ | 0.40 ± 0.81^£^ | -0.37 ± 1.07^§,£^ | *H* = 18.11 | **<0.001** |
| Domain: Language |  |  |  |  |  |
| Semantic fluency | 1.27 ± 0.66^¥,§^ | 0.46 ± 0.78^¥^ | -0.12 ± 0.94^§^ | *H* = 30.14 | **<0.001** |
| Boston Naming Test | 0.00 ± 1.00^§^ | -0.10 ± 0.77 | -0.97 ± 1.42^§^ | *H* = 13.41 | **0.001** |
| Domain: Memory |  |  |  |  |  |
| Delayed recall PANDA | 0.00 ± 1.00^§^ | -0.46 ± 1.37^£^ | -3.70 ± 2.23^§,£^ | *H* = 34.56 | **<0.001** |
| Delayed recall MMSE | 0.00 ± 1.00 | 0.49 ± 0.76^£^ | -0.71 ± 1.20^£^ | *H* = 15.74 | **<0.001** |
| Domain: Visual spatial abilities |  |  |  |  |  |
| Pentagons MMSE | 0.00 ± 1.00^§^ | -0.03 ± 1.05^£^ | -1.79 ± 2.01^§,£^ | *H* = 23.26 | **<0.001** |
| Cubes PANDA | 0.00 ± 1.00^§^ | 0.19 ± 0.99^£^ | -1.02 ± 1.17^§,£^ | *H* = 16.28 | **<0.001** |
| Overall cognition z-score | 0.32 ± 0.43^¥,§^ | 0.04 ± 0.36^¥,£^ | -1.08 ± 0.52^§,£^ | *H* = 53.309 | **<0.001** |
| CFQ | 23.80 ± 12.68 | 27.15 ± 10.55 | 26.26 ± 12.42 | *H* = 1.470 | 0.480 |
| CFQ z-score | 0.00 ± 1.53 | -0.40 ± 1.27 | -0.30 ± 1.50 | *H* = 1.470 | 0.480 |
| ISAcog | 0.32 ± 1.47^§^ | 0.44 ± 1.35^£^ | -0.78 ± 1.51^§,£^ | *H* = 9.770 | **0.008** |

Abbreviations: PD, Parkinson’s disease; PD-NC; PD with normal cognition; PD-MCI, PD with mild cognitive impairment; MMSE, Mini Mental State Examination; PANDA, Parkinson Neuropsychometric Dementia Assessment; CFQ, Cognitive Failures Questionnaire; ISAcog, impaired self-awareness of cognitive deficits.

Post-hoc U-test (p<0.00167): ^¥^ significant difference between controls and PD-NC; ^§^ significant difference between controls and PD-MCI; ^£^ significant difference between PD-NC and PD-MCI.
