# Supplementary material for: Impaired self-awareness of cognitive deficits in Parkinson's disease relates to cingulate cortex dysfunction
Source: Psychol Med. 2021 Sep 23;53(4):1244–53. doi: 10.1017/S0033291721002725 (PMC10009405; doi:10.1017/S0033291721002725)
Supplement: Supplementary file 1 [file S0033291721002725sup.zip › S0033291721002725sup004.docx]

|  | **All PD patients**  **(N=63)**  **r** | **PD-MCI**  **(N=23)**  **r** |
| --- | --- | --- |
| Correlation of ISAcog with: |  |  |
| age | -0.111 | 0.358 |
| Education | 0.141 | 0.184 |
| Disease duration | -0.130 | -0.124 |
| UPDRS-III | -0.228 | -0.360 |
| LEDD | -0.083 | -0.223 |
| BDI-2 | 0.300^*^ | 0.088 |
| Executive functions | 0.412^***^ | 0.128 |
| Memory | 0.287^*^ | 0.179 |
| Language | 0.279^*^ | 0.408 |
| Visual-spatial abilities | 0.354^***^ | 0.189 |
| Attention | 0.199 | -0.228 |

**Table S4:** Results of Spearman correlation analysis between ISAcog and clinical data in all PD patients and PD-MCI.

Abbreviations: PD, Parkinson’s disease; PD-MCI: patient with mild cognitive impairment; ISAcog, impaired self-awareness of cognitive deficits; UPDRS-III, Unified Parkinson’s disease Rating Scale; LEDD, Levodopa equivalent daily dose; BDI-2, Beck Depression Inventory-2.

^*^ p<.05, ^**^ p<.01, ^***^p<.001
